# Stroke Prevention in Patients with Atrial Fibrillation

**DOI:** 10.19102/icrm.2018.090405

**Published:** 2018-04-15

**Authors:** Peter R. Kowey


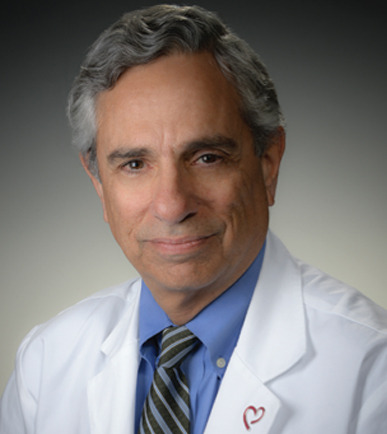


It ain’t what you don’t know that gets you into trouble. It’s what you know for sure that just ain’t so (Mark Twain).

Those of us in the academic community ardently believed that the advent of the new oral anticoagulants would have a revolutionary impact on the care of patients with atrial fibrillation. It has taken a while, and the “revolution” has come to look more like an “evolution,” but market statistics now substantiate a steady and impressive uptake of several of the new agents by health care providers around the globe. With increased use, however, has come the inevitable realization that we don’t know as much as we should about the everyday use of these very important drugs. In essence, they are not as simple to implement as we had presumed. Multiple knowledge gaps have been identified that need to be addressed to optimize their application. Fortunately, several thought leaders have taken up the challenge and have designed and participated in clinical trials aiming to plug the holes that exist.

In this issue of *The Journal of Innovations in Cardiac Rhythm Management*, three articles are presented, each addressing an important issue related to the prevention of stroke in patients with atrial fibrillation. Ruff^1^ takes on the important question of reversal agents, one of the very first topics patients and referring doctors ask about when a new anticoagulant is proposed. While the news he presents is encouraging, there is clearly much more to be done to make these agents available and then to figure out how to use them most efficiently and appropriately in the community.

Given the relatively short half-life of these new drugs in comparison with vitamin K antagonists, it would be logical to consider how they might be used intermittently instead of constantly, in order to reduce their risks and costs. However, before such a strategy can be recommended or propagated, comprehensive trials of the safety and efficacy of such an approach are essential. Matos et al.^2^ provide leadership in this regard and thoughtfully review our current state of knowledge regarding periodic therapy.

Finally, we know that many patients cannot or do not wish to be treated with an oral anticoagulant. To this effect, Chava et al.^3^ share their perspective on the utility of mechanical devices as an alternative for stroke protection in carefully selected patients.

We thank the authors for their thoughtful and balanced approach to important issues and look forward to continuing efforts to learn more about this exciting new class of drugs, with the ultimate goal of improving their safety and acceptability in preventing the catastrophe of stroke in as many of our patients as possible.

Sincerely,


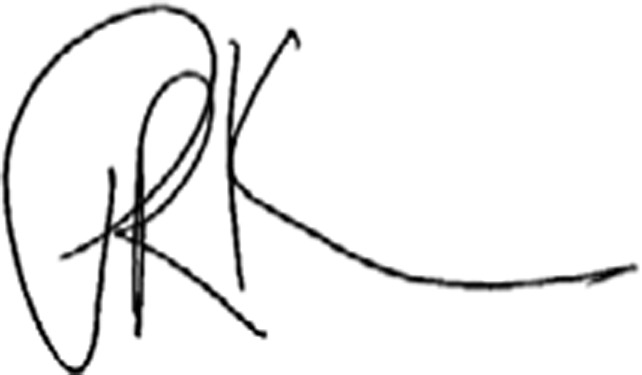


Peter R. Kowey, FACC, FAHA, FHRS

Professor of Medicine and Clinical Pharmacology

Jefferson Medical College

Chairman of Cardiology, Emeritus

Lankenau Heart Institute

W. W. Smith Chair Cardiovascular Research

Lankenau Institute for Medical Research

Wynnewood, PA 19096
